# Spatial profiling of the placental chorioamniotic membranes reveals upregulation of immune checkpoint proteins during Group B *Streptococcus* infection in a nonhuman primate model

**DOI:** 10.3389/fcimb.2023.1299644

**Published:** 2024-01-04

**Authors:** Gygeria Manuel, Michelle Coleman, Austyn S. Orvis, Jeff Munson, Amanda Li, Raj P. Kapur, Miranda Li, Edmunda Li, Blair Armistead, Lakshmi Rajagopal, Kristina M. Adams Waldorf

**Affiliations:** ^1^ Department of Obstetrics and Gynecology, University of Washington, Seattle, WA, United States; ^2^ Morehouse School of Medicine, Atlanta, GA, United States; ^3^ Center for Global Infectious Disease Research, Seattle Childrens Research Institute, Seattle, WA, United States; ^4^ Department of Psychiatry and Behavioral Sciences, University of Washington, Seattle, WA, United States; ^5^ Department of Biology, Case Western Reserve University, Cleveland, OH, United States; ^6^ Department of Laboratory Medicine and Pathology, Seattle Children’s Hospital and University of Washington, Seattle, WA, United States; ^7^ School of Medicine, University of Washington, Seattle, WA, United States; ^8^ Department of Global Health, University of Washington, Seattle, WA, United States

**Keywords:** Group B *Streptococcus*, pregnancy, placenta, amnion, chorion, decidua, immune checkpoint

## Abstract

**Background:**

Preterm birth is a leading cause of neonatal mortality, which is often complicated by intrauterine infection and inflammation. We have established a nonhuman primate model of Group B *Streptococcus* (GBS, *Streptococcus agalactiae*) infection-associated preterm birth. Immune checkpoints are modulators of the immune response by activating or suppressing leukocyte function and are understudied in preterm birth. The objective of this study was to spatially profile changes in immune protein expression at the maternal-fetal interface during a GBS infection with a focus on immune checkpoints.

**Methods:**

Twelve nonhuman primates (pigtail macaques, *Macaca nemestrina*) received a choriodecidual inoculation of either: 1) 1-5 X 10^8^ colony forming units (CFU) of hyperhemolytic/hypervirulent GBS (GBSΔ*covR*, N=4); 2) an isogenic/nonpigmented strain (GBS Δ*covR*Δ*cylE*, N=4); or, 3) saline (N=4). A Cesarean section was performed at preterm labor or 3 days after GBS infection or 7 days after saline inoculation. Nanostring GeoMx® Digital Spatial Profiling technology was used to segment protein expression within the amnion, chorion, and maternal decidua at the inoculation site using an immuno-oncology panel targeting 56 immunoproteins enriched in stimulatory and inhibitory immune checkpoint proteins or their protein ligands. Statistical analysis included R studio, Kruskal-Wallis, Pearson and Spearman tests.

**Results:**

Both inhibitory and stimulatory immune checkpoint proteins were significantly upregulated within the chorioamniotic membranes and decidua (VISTA, LAG3, PD-1, CD40, GITR), as well as their ligands (PD-L1, PD-L2, CD40L; all p<0.05). Immunostaining for VISTA revealed positive (VISTA+) cells, predominantly in the chorion and decidua. There were strong correlations between VISTA and amniotic fluid concentrations of IL-1β, IL-6, IL-8, and TNF-α (all p<0.05), as well as maternal placental histopathology scores (p<0.05).

**Conclusion:**

Differential regulation of multiple immune checkpoint proteins in the decidua at the site of a GBS infection indicates a major perturbation in immunologic homeostasis that could benefit the host by restricting immune-driven pathologies or the pathogen by limiting immune surveillance. Protein expression of VISTA, an inhibitory immune checkpoint, was upregulated in the chorion and decidua after GBS infection. Investigating the impact of innate immune cell expression of inhibitory immune checkpoints may reveal new insights into placental host-pathogen interactions at the maternal-fetal interface.

## 1 Introduction

Group B Streptococci (GBS) are commensal gram-positive bacteria that reside in the gastrointestinal and lower reproductive tracts of approximately 18% of pregnant women (Russell et al., 2017a). In pregnancy, GBS can ascend into the uterus causing infection of the placenta and fetus resulting in preterm birth, stillbirth, invasive neonatal disease and neurodevelopmental deficits in the neonate ([[NoAuthor]]; Bianchi-Jassir et al., 2017; Russell et al., 2017b; Seale et al., 2017b; Horváth-Puhó et al., 2021). In 2015, it was estimated that GBS was a leading cause of approximately 3.5 million preterm births and 409,000 cases of adverse maternal, fetal or infant outcomes (Seale et al., 2017a). The pathogenesis of invasive GBS infections and how innate immune defenses prevent or fail to control a GBS placental infection are poorly understood but may be leveraged to improve maternal-fetal health.

We have established a nonhuman primate model in which GBS are inoculated into the choriodecidual space of the lower uterine segment where ascending bacteria are first thought to contact the placenta (Adams Waldorf et al., 2011a; Vanderhoeven et al., 2014; McAdams et al., 2015; Boldenow et al., 2016; Coleman et al., 2020; Weed et al., 2020; Coleman et al., 2021). The virulence of the GBS strain is paramount for the outcome of a choriodecidual infection in that strains of higher virulence have a greater likelihood of ascension into the uterus, placental invasion and preterm birth or stillbirth (Whidbey et al., 2013; Boldenow et al., 2016; Vornhagen et al., 2016; Armistead et al., 2019; Brokaw et al., 2021; Coleman et al., 2021; Furuta et al., 2022; Huebner et al., 2022). A key GBS virulence factor is the ornithine rhamnolipid pigment (β-hemolysin), which imparts hemolytic activity, which is produced by gene products in the *cyl* operon (Spellerberg et al., 1999; Pritzlaff et al., 2001; Whidbey et al., 2013). In our nonhuman primate model, we have previously demonstrated that overexpression of β-hemolysin is associated with adverse perinatal outcomes, such as bacterial invasion and preterm labor. However, spatial immune events along the path of invasive bacterial infection from the decidua through the chorioamniotic membranes are not well-characterized or understood.

Interrogation of the spatial immune protein expression in the placenta also presents the opportunity to evaluate the role of the amnion, chorion, and decidua in activating and restraining the inflammatory response. The maternal-fetal interface defined by the maternal decidua adjacent to the fetal chorion plays a key role in maintaining fetal tolerance. Immune checkpoint proteins act to inhibit or activate various aspects of T cell function (proliferation, activation) and have been studied extensively in oncology, but recent evidence indicates that they may play a role in adverse pregnancy outcomes, such as preeclampsia, recurrent pregnancy loss and villitis of unknown etiology (Ozen et al., 2018; Miko et al., 2019; Shahi et al., 2021; Esparvarinha et al., 2023). Whether their placental expression may be regulated in the course of acute chorioamnionitis to counteract the deleterious effects of the inflammatory cascade is unknown.

Our study objective was to spatially profile immune proteins in the placental chorioamniotic membranes and decidua following an experimental GBS choriodecidual infections in a nonhuman primate model. We hypothesized that an early GBS infection induces expression of immune checkpoint proteins in the chorioamniotic membranes to counteract the cytokine and chemokine response that has the potential for recruiting T cells into the membranes to maintain tolerance of the fetal allograft. Understanding the balance of inflammatory versus tolerance-promoting immune proteins in the amnion, chorion and decidua would be helpful to gain insight into how the placenta responds to infection.

## 2 Methods

### 2.1 Ethics approval

All animal experiments were carried out in strict accordance with the recommendations in the Guide for the Care and Use of Laboratory Animals of the National Research Council and the Weatherall report, “The use of non-human primates in research.” The University of Washington Institutional Animal Care Use Committee approved the protocol (Permit Number: 4165-01, last approved 02/09/2021). All surgery was performed under general anesthesia and all efforts were made to minimize suffering.

### 2.2 Nonhuman primate model study design

**Figure 1:**
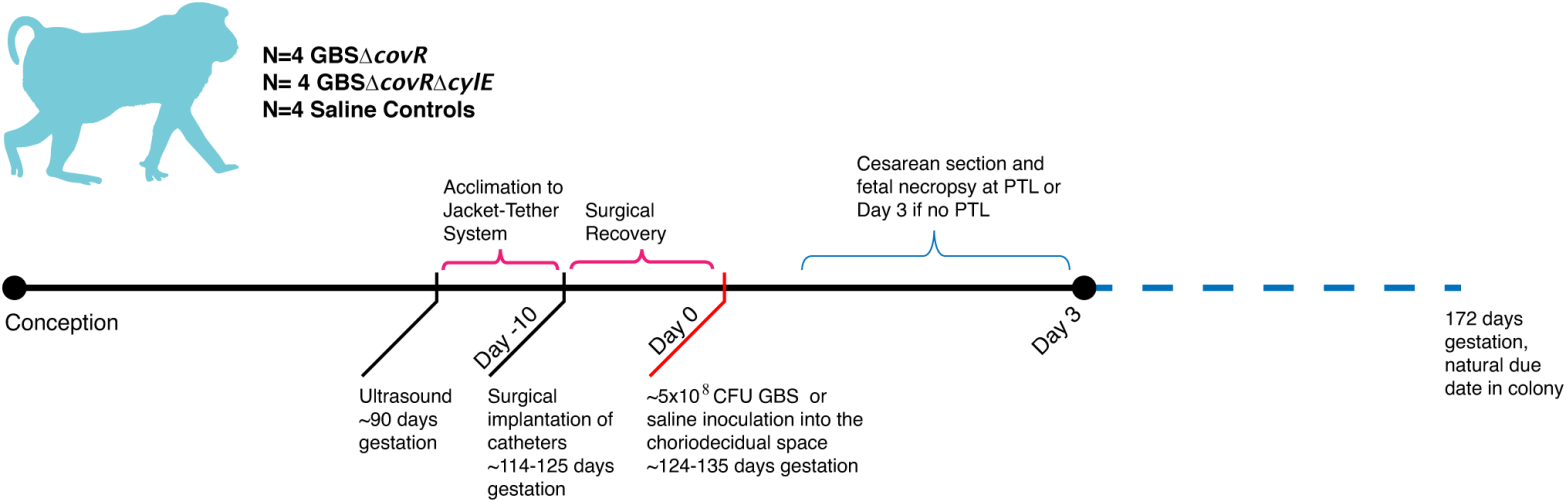
This figure shows the study design. The x-axis reflects both days with respect to inoculation and is not to scale. The approximate days since conception are indicated in text. There were four nonhuman primates (NHP) in each experimental group: saline, GBSΔ*covR*, and GBSΔ*cov*RΔ*cyl*E.

In this study, eleven pregnant NHP (*Macaca nemestrina*) had catheters surgically implanted via laparotomy into the maternal femoral vein, amniotic fluid, and choriodecidual interface in the lower uterine segment (between uterine muscle and fetal membranes, external to amniotic fluid). Another pregnant NHP was an uncatheterized control. Animals were selected to undergo choriodecidual inoculation with one of two different GBS strains: i.e., approximately 1-5 x 10^8^ colony forming units (CFU) of either GBS COH1Δ*covR* (N=4; hyperpigmented strain) or GBS COH1Δ*covRΔcylE* (N=4; isogenic, nonpigmented strain). The study design is shown in **Figure 1** with details regarding inoculum CFU, gestational age at inoculation, the interval from inoculation to delivery, and the inoculum dose in **Supplementary Table 1**. Results obtained from these animals were compared to saline controls (N=4; choriodecidual and amniotic fluid saline inoculations and one non-catheterized control) that were performed previously (Adams Waldorf et al., 2011a; Boldenow et al., 2016).

### 2.3 Sample collection and processing

Preterm labor (PTL) was defined as progressive cervical dilation associated with increased uterine activity (>10,000 mmHg•sec/hr sustained over at least 2 hours). Cesarean section was performed at the following endpoints to allow for tissue collection following: 1) preterm labor, 2) three days after GBS inoculation if no preterm labor was observed, or 3) 7-days after saline inoculation (Boldenow et al., 2016). A 3-day endpoint to assess the effects of GBS on placental tissues and fetal injury was chosen to study the earliest events in the pathway of infection/inflammation associated preterm birth. The 7-day endpoint for saline controls was chosen at the inception of our research program and provides a close gestational age match for the current study. After the Cesarean section, fetuses were euthanized by barbiturate overdose followed by exsanguination and fetal necropsy. Clinical outcomes are summarized in **Supplementary Table 2**.

Amniotic fluid (AF) was sampled frequently before (-24 and -0.25 hours) and after GBS inoculation (+0.75, +6, +12, +24 hours and then every 12 hours until repeat C-section for fetal necropsy). For cytokine and prostaglandin (PG) analysis, samples of AF were collected in EDTA (BD biosciences, San Jose, California, USA) tubes. The peak AF cytokine concentration was identified at the sampling time point where each individual cytokine was highest in each animal post-inoculation. Next, the peak AF cytokine concentration was correlated with immunoprotein expression in each placental region of interest (ROI) targeted in this experiment.

Intraamniotic pressure was continuously recorded using an implanted amniotic fluid pressure catheter (SPR-524, ADInstruments, Colorado Springs, Colorado, USA) and digitized with a Powerlab System (ADInstruments) connected to a desktop computer. Amniotic fluid pressure signals were processed using custom software to eliminate noise due to respiration or position changes. The area under each contraction (mmHg·sec/hr) was summed for each hour allowing calculation of the hourly contraction area, a measure of uterine activity. Finally, we calculated the mean hourly contraction area [(mmHg x sec)/hr); HCA] over a 24-hour period. We used the peak mean HCA for each animal to test correlations between uterine activity and immunoprotein antigen counts in each placental ROI.

### 2.4 GBS strains and bacterial enumeration

The GBS hyperhemolytic/hyperpigmented Δ*covR* and isogenic nonpigmented Δ*covR*Δ*cylE* strains were derived from wild type (WT) GBS COH-1, an ST-17 clone belonging to capsular serotype III which was obtained from an infected newborn and were previously described (Boldenow et al., 2016). Routine cultures of GBS were grown in tryptic soy broth (TSB) or tryptic soy agar (TSA, Difco Laboratories) at 37°C in 5% CO2. For inoculations in the NHP model, GBS strains were grown to mid-log phase (O.D.600 = 0.5) and approximately 1-5 X 10^8^ CFU in 1mL PBS was inoculated into the choriodecidual space, as described previously (Kuypers et al., 1989). For bacterial enumeration, AF (200 µL) from each sampling timepoint was serial diluted and 10-fold dilutions were plated on TSA, incubated overnight at 37°C, 5% CO2 and enumerated to determine bacterial invasion.

### 2.5 Confirmation of GBS from infected animals

The hyperpigmented GBSΔcovR strain has an orange color when plated on TSA and nonpigmented GBS strains are white in color (Whidbey et al., 2013). We also note that Δ*covR* and Δ*covR*Δ*cylE* strains of GBS are spectinomycin-resistant because the gene *covR* was replaced with a gene conferring spectinomycin resistance in these strains (Jiang et al., 2005). To confirm that the GBS strains recovered from infected NHP were the correct strains, a few colonies obtained from each sampled tissue and fluid per experiment were patched on selective medium (i.e., TSA containing spectinomycin), and the level of CAMP factor activity was tested on sheep blood agar plates with the inoculum strain included in parallel.

### 2.6 Amniotic fluid processing

Amniotic fluid (AF) was sampled before (−24 and −0.25 h) and after pathogen inoculation (+0.75, +6, +12, +24 h and then every 12 h until Cesarean section for fetal necropsy) to culture for GBS and assay for inflammatory mediators. For cytokine analysis, samples of amniotic fluid were collected in EDTA tubes. Samples were centrifuged for 5 min at 1,200 rpm immediately after collection and the supernatant was frozen and stored at −80°C. Tissues were weighed at necropsy, homogenized in sterile PBS and 10-fold serial dilutions were plated on TSA to count bacterial colonies; plates were incubated overnight at 37°C, 5% CO2 and enumerated as described (Nizet et al., 1997; Winram et al., 1998). 

### 2.7 Cytokine measurement

Cytokine concentrations were determined using Luminex multiplex cytokine kits (Millipore Sigma, Burlington, MA), following manufacturer’s instructions. I-TAC, IFN-γ, IL-1β, IL-6, IL-8, IP-10, MCP-1, MIG and TNF-α were detected. Prostaglandin E2 (PGE2) and Prostaglandin F2-alpha (PGF2α) were determined using commercially available human EIA kits (Cayman Chemical, Ann Arbor, Michigan, USA) following manufacturer’s instructions. Cytokine data was incomplete for two animals due to insufficient samples.

### 2.8 Nanostring GeoMx® digital spatial profiling (DSP)

**Figure 2:**
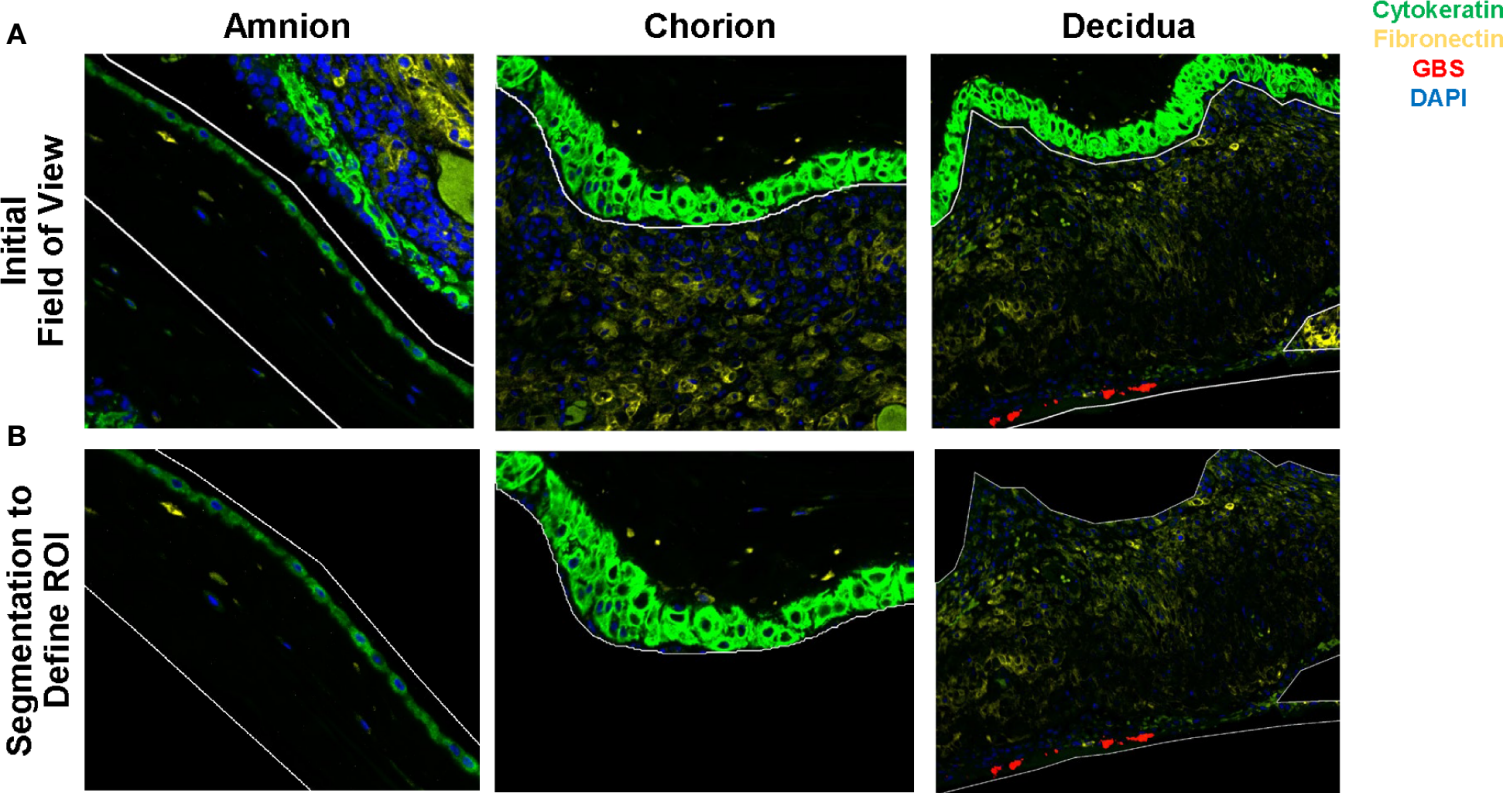
Segmentation of the amnion, chorion and decidua was performed using the Nanostring GeoMx® Digital Spatial Profiling platform. Row **(A)** demonstrates the initial field of view using fluorescent microscopy and the regions of interest drawn to segment the amnion, chorion, and decidua. Row **(B)** demonstrates the segmented tissue regions prior to UV cleavage and capillary collection of the probes from the area of interest.

GeoMx digital spatial profiling (DSP) was performed at NanoString Technologies in Seattle, WA. Formalin-fixed, paraffin-embedded placental sections from animals from each group were incubated with fluorescent probes and eventual multiplex cocktail of primary antibodies with photocleavable oligonucleotides (i.e., a validated DSP human-immune oncology protein panel; NanoString Technologies). Fluorescent antibodies used to highlight features of the histology and draw the ROIs included: 1) anti-pan cytokeratin-Alexa Fluor 488 (Pan-CK, clone AE1/AE3; Novusbio, cat# NBP2-33200AF488); 2) anti-fibroblast activation protein-Alexa-Fluor 594 (FAP, clone SP325; Abcam; cat# ab311827); 3) SYTO 83 for nuclei visualization (ThermoFisher, Waltham, Ma); and 4) anti-GBS-Alexa Fluor 647 (Abcam; cat# ab53584). Sections were magnified 20 times, and ROIs comprising the decidua, chorion, and amnion from each animal were selected based on tissue morphology (**Figure 2**). The 56 immune protein panel of oligoconjugated antibodies were applied to the tissue. Each ROI was then exposed to UV illumination with a double digital mirror device molecule, which cleaved the DNA oligonucleotides into the aqueous layer above the tissue slice. The oligonucleotides in the eluent were collected via microcapillary aspiration and transferred to an individual well of a microtiter plate. Oligonucleotides were then hybridized to Nanostring nCounter optical barcodes to permit *ex situ* digital counting of each analyte. Briefly, hybridization of oligonucleotides to optical barcodes were performed at 65°C in a thermocycler. After hybridization, samples were processed using the nCounter prep station and digital analyzer. Data were normalized to technical controls and the S6 housekeeping protein. To generate signal/noise ratios, data were calculated relative to isotype controls.

### 2.9 Nanostring GeoMx® 56-antibody immune panel

The 56 immune protein panel was designed to target antigens from common immunology and immuno-oncology proteins. These proteins include VISTA, TIM-3, TGFB-1, SMA, S100B, PTEN, PR, PD-L2, PD-L1, PD-1, PanCk, OX40L, NY-ESO-1, MART1, LAG3, Ki67, IDO1, ICOS, HLA-DR, Her2/ErbB2, GZMB, GITR, γδ-TCR, FOXP3, Fibronectin, Fapα, ERα, EpCAM, CTL-A4, CD86, CD80, CD8, CD68, CD66b, CD56, CD45RO, CD45, CD44, CD40L, CD40, CD4, CD34, CD3, CD27, CD25, CD20, CD163, CD14, CD127, CD11c, Beta-2-microglobulin, BCL-2, B7-H3, ARG1, and 4-1BB CD137. In addition to these 56 immune proteins, the panel included six control antibodies: mouse IgG1, mouse IgG2a, rabbit IgG, GAPDH, Histone H3, and ribosomal S6. Antibodies against stimulatory checkpoint proteins and protein ligands included CD27, CD40, CD40L, CD137, OX40L, GITR and ICOS. Inhibitory checkpoint antibodies in the panel targeted B7-H3, CTLA-4, IDO1, LAG-3, PD-1, PD-L1, PD-2, TIM-3, ARG1 and VISTA.

### 2.10 DSP normalization

Data was normalized using the Ribosomal S6 protein, which correlated more consistently with the tissue area in each region (amnion, chorion, decidua) than GAPDH or Histone H3. Plots displaying correlation of housekeeping proteins with tissue area are shown in Supplementary data (**Supplementary Figure 1**).

### 2.11 Immunohistochemistry validation

Immunohistochemistry staining was performed using two antibodies, VISTA and MPO, on placental chorioamniotic tissues. Tissues were stained with rabbit anti-human VISTA (D1L2G™) XP® 1:200 dilution (catalog #64953, Cell Signaling, Danvers, Massachusetts, USA) antibody, rabbit anti-human MPO 1:100 dilution (catalog# PA5-16672, ThermoScientific, Waltham, MA) or rabbit IgG (catalog # AB-105-C, R&D Systems, Minneapolis, MN) isotype controls. Briefly, slides were prepared from formalin fixed and paraffin embedded specimens of placental tissue. Dewaxing was performed in a xylene series and antigen retrieval performed in a citrate buffer with heat for 10 minutes. Following antigen retrieval, the slides were blocked with 10% normal goat serum. Staining was performed using the BOND automated IHC Stainer (Leica, Wetzlar, Germany). Immunohistochemical cell staining was quantified using Visiopharm software (Visopharm, Hørsholm, Denmark). Regions of interest (ROI) in the decidua were drawn manually and the percent positive staining was calculated.

### 2.12 Placental Redline scoring

Placentas were scored by a pediatric pathologist (RK), who was blinded to treatment according to the Redline criteria (Redline et al., 2003).

### 2.13 Statistical analysis

A p-value less than 0.05 was considered significant. Analyses were performed using R studio. Statistical tests included the Kruskal-Wallis and Pearson’s correlation. The hypothesis focused on differences between the GBSΔ*covR* and saline groups, therefore, correction for multiple comparisons was not performed.

## 3 Results

### 3.1 Digital spatial profiling (DSP) in GBS model demonstrates differentially expressed immune checkpoints at the site of infection

**Figure 3:**
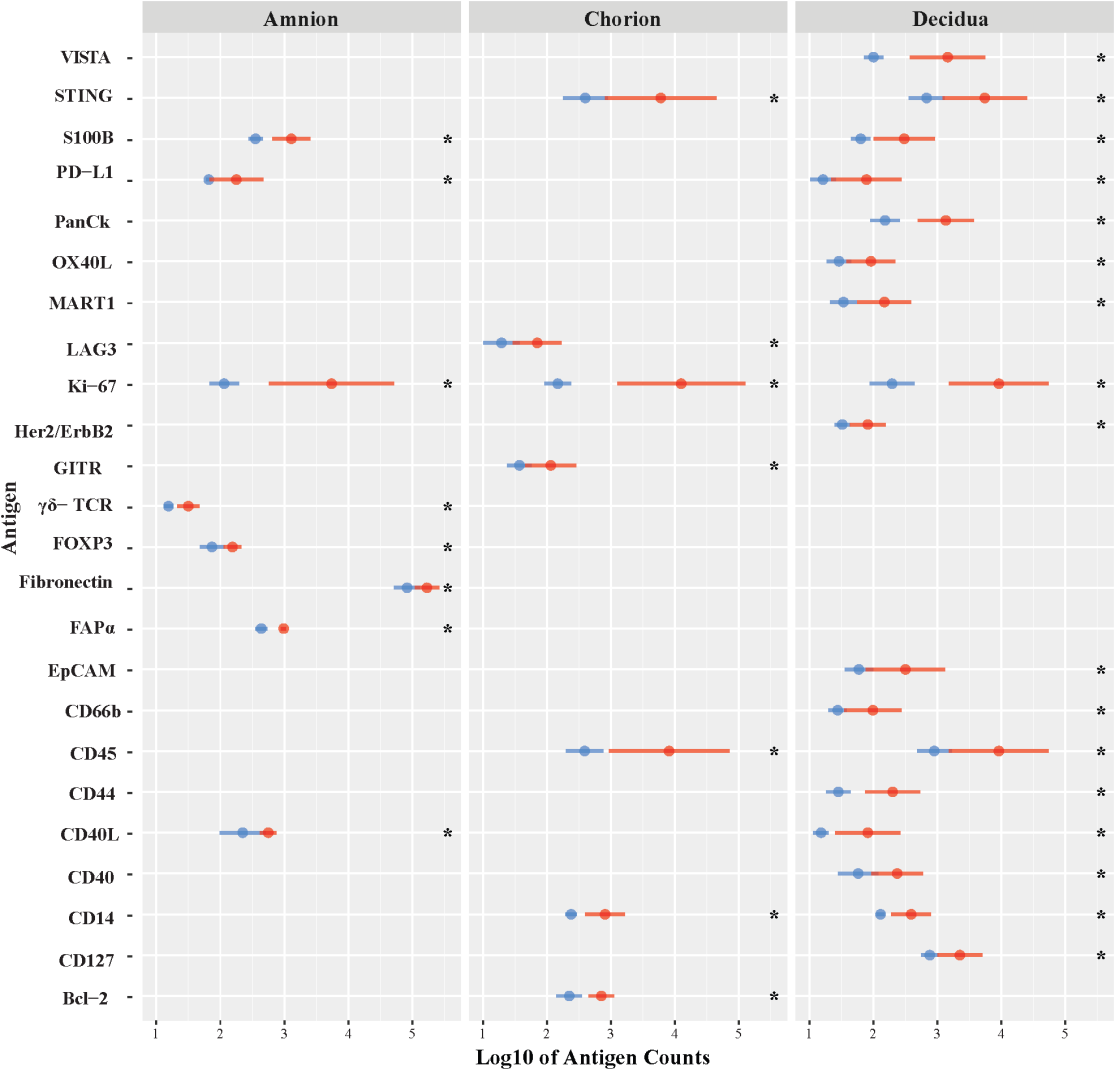
Differential expression of DSP antigens by Region of Interest for GBSΔ*covR v*ersus Saline. The log10 S6 normalized antigen counts for GBSΔ*covR* (red) and saline controls (blue) are shown on the x-axis by region of the chorioamniotic membranes with the mean (dot) and +/- 1 standard deviation (bar). Although the entire GeoMx panel included 56 antigens, we show only the 24 antigens (y-axis) that were significantly different between the GBSΔ*covR* and saline controls in either the amnion, chorion or decidua using Kruskal-Wallis (*, p <0.05).

First, we evaluated differences in immunoprotein expression within the amnion, chorion, and decidua at the site of the GBS infection for the primary comparison between GBSΔ*covR* and saline controls (**Figure 3**). All immunoproteins found to be significantly differentially expressed were upregulated in the GBSΔ*covR* group versus saline controls. The greatest number of upregulated immunoproteins was in the decidua with a lesser number of differentially upregulated proteins in the amnion and chorion (**Figure 3**; **Table 1**). Inhibitory immune checkpoint proteins/ligands were significantly upregulated in all ROIs including VISTA (decidua), LAG-3 (chorion), and PD-L1 (ligand for PD-1, amnion and decidua; all p<0.05). In addition to overexpression of inhibitory immune checkpoint proteins, many stimulatory checkpoint proteins/ligands that activate B and T cells (OX40L, CD40, CD40L, GITR) were significantly upregulated (all p<0.05). Across all ROIs, Ki67 was also significantly differentially upregulated indicating widespread cell cycle activity and/or proliferation (p<0.05, each ROI).

**Table 1:** Differentially expressed immunoproteins in the Amnion, Chorion and Decidua at the site of a GBSΔ*covR* inoculation.

Antigen	Mean Expression for Saline Controls	Mean Expression for GBSΔ*covR*	Pooled Standard Deviation	Standard Differences between GBSΔ*covR* and Saline Controls	Kruskal-Wallis Statistic	Kruskal-Wallis p-value
	Amnion					
FAPα	2.64	2.99	0.07	5	5.33	0.02
S100B	2.55	3.11	0.23	2.43	5.33	0.02
γ-TCR	1.19	1.5	0.13	2.38	4.08	0.04
Ki-67	2.06	3.74	0.71	2.37	5.33	0.02
FOXP3	1.87	2.19	0.17	1.88	4.08	0.04
Fibronectin	4.92	5.23	0.2	1.55	4.08	0.04
CD40L	2.35	2.75	0.27	1.48	4.08	0.04
PD-L1	1.82	2.25	0.3	1.43	5.33	0.02
	Chorion					
Ki-67	2.17	4.1	0.72	2.68	5.33	0.02
Bcl-2	2.35	2.85	0.2	2.5	5.33	0.02
CD14	2.38	2.91	0.23	2.3	5.33	0.02
CD45	2.59	3.91	0.7	1.89	4.08	0.04
STING	2.6	3.78	0.66	1.79	4.08	0.04
LAG3	1.29	1.85	0.33	1.7	5.33	0.02
GITR	1.57	2.06	0.31	1.58	4.08	0.04
	Decidua					
Ki-67	2.29	3.96	0.6	2.78	5.33	0.021
PanCk	2.18	3.13	0.35	2.71	5.33	0.021
VISTA	2	3.16	0.43	2.7	5.33	0.021
CD44	1.45	2.3	0.33	2.58	5.33	0.021
CD14	2.11	2.59	0.22	2.18	5.33	0.021
CD40L	1.18	1.91	0.37	1.97	5.33	0.021
MART1	1.53	2.17	0.33	1.94	5.33	0.021
S100B	1.8	2.48	0.36	1.89	5.33	0.021
Her2/ErbB2	1.51	1.91	0.22	1.82	4.08	0.043
CD127	0.88	3.35	0.26	1.81	4.08	0.043
STING	2.83	3.74	0.51	1.78	40.8	0.043
CD45	2.95	3.96	0.58	1.74	4.08	0.043
CD40	1.76	2.37	0.36	1.69	4.08	0.043
CD66b	1.44	1.99	0.33	1.67	4.08	0.043
OX40L	1.46	1.96	0.3	1.67	5.33	0.021
PD-L1	1.21	1.89	0.41	1.66	4.08	0.043
EpCAM	1.77	2.5	0.47	1.55	4.08	0.043

This table lists the significantly differentially expressed immunoproteins within the amnion, chorion and decidua for the GBSΔ*covR* versus saline control group. Within each region of interest, the antigens were listed in descending order by the standard difference between GBSΔ*covR* and saline controls.

**Figure 4:**
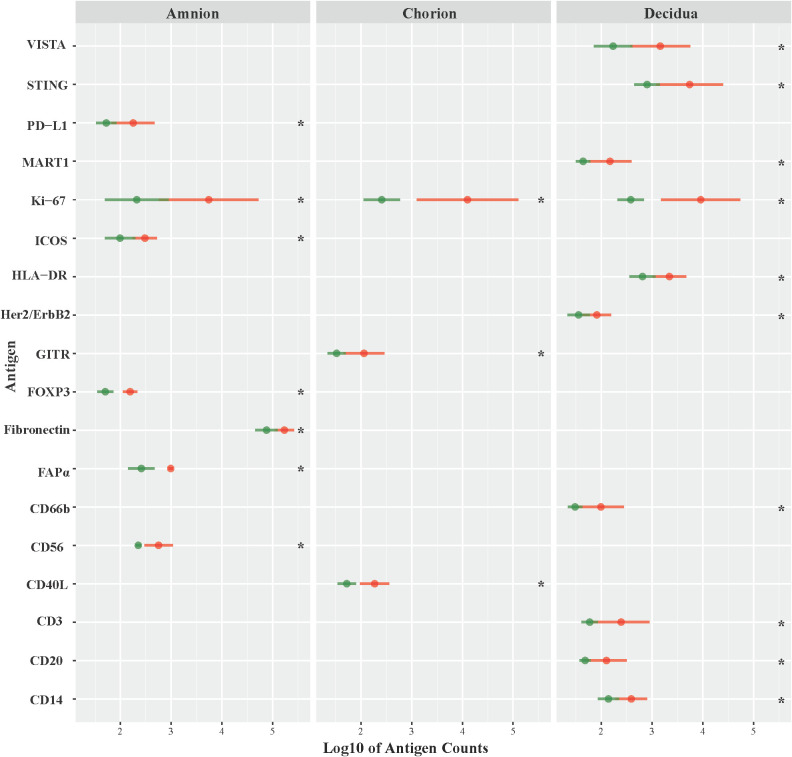
Differential expression of DSP antigens by Region of Interest GBSΔ*covR* versus GBSΔ*cov*RΔ*cyl*E. The log10 S6 normalized antigen counts for GBSΔ*covR* (red) and GBSΔ*covR*Δ*cylE* (green) are shown on the x-axis by region of the chorioamniotic membranes with the mean (dot) and +/- 1 standard deviation (bar). Although the entire GeoMx panel included 56 antigens, we show only the 18 antigens (y-axis) that were significantly different between the GBSΔ*covR* and saline controls in either the amnion, chorion or decidua using Kruskal-Wallis (*, p <0.05).

Next, we investigated the role of the GBS β-hemolysin in directing the shift in immune checkpoint expression by comparing immunoprotein expression in GBSΔ*covR* (overexpressing β-hemolysin) versus GBSΔ*covR*Δ*cylE* (lacking β-hemolysin) exposed tissues. In this comparison, the profile of immunoprotein expression was very similar to that obtained when comparing GBSΔ*covR* versus saline controls, which underscored the importance of the hemolysin to GBS virulence and induction of host immunologic response (**Figure 4**; **Table 2**). In both comparisons, there was overexpression in GBSΔ*covR* of immune checkpoint proteins/ligands PD-L1 (amnion), GITR (chorion), and VISTA (decidua). In contrast, when antigen counts were analyzed for the comparison of GBSΔ*covR*Δ*cylE* versus saline controls, only the gamma-delta T cell receptor (γδ-TCR) antigen was significantly differentially expressed in the GBSΔ*covR*Δ*cylE* group (p<0.05) (**Supplementary Table 3**).

**Table 2:** Differentially expressed immunoproteins in Amnion, Chorion, and Decidua for each experimental group contrast.

Significant DSP Antigen	GBSΔ*covR* vs. Control ​			GBSΔ*covR* vs GBSΔ*covR*Δ*cylE*		
	Amnion	Chorion	Decidua	Amnion	Chorion	Decidua
Bcl-2		X				
CD3						X
CD14		X	X			X
CD20						X
CD40			X			
CD40L	X		X		X	
CD44			X			
CD45		X	X			
CD56				X		
CD66b			X			X
CD127			X			
EpCAM			X			
FAPα	X			X		
Fibronectin	X			X		
FOXP3	X			X		
γδ-TCR	X					
GITR		X			X	
Her2/ErbB2			X			X
HLA-DR						X
ICOS				X		
Ki-67	X	X	X	X	X	X
LAG3		X				
MART1			X			X
OX40L			X			
PanCk			X			
PD-L1	X		X	X		
STING		X	X			X
S100B	X		X			
VISTA			X			X

This table shows the differentially expressed immunoproteins for each experimental group contrast. An X indicates a significant (p<0.05) comparison within a specific region of interest (amnion, chorion, decidua).

**Figure 5:**
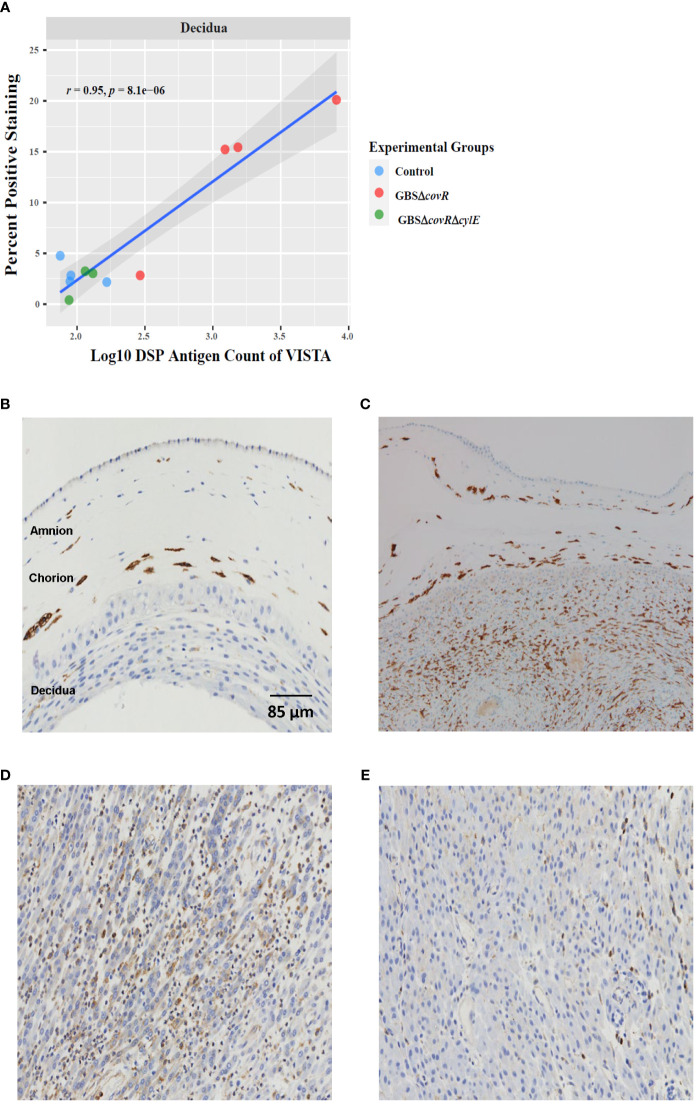
VISTA correlation and expression by DSP and Immunohistochemistry. **(A)** shows the relationship between log_10_DSP antigen counts and percent positive VISTA immunostaining (DAB, brown stain) out of the total area stained (DAB + haematoxylin blue stain) for GBSΔ*covR* (red) and GBSΔ*covR*Δ*cylE* (green), and controls (blue) in the decidua. VISTA **(B)** and CD163 **(C)** immunostaining of the amnion, chorion and decidua are shown to demonstrate morphologic overlap between the staining profiles in a saline control. Immunohistochemistry targeting VISTA in the decidua is shown for GBSΔ*covR* **(D)** and GBSΔ*covR*Δ*cylE* **(E)** groups to show the cellular influx of VISTA+ cells in the GBSΔ*covR* group.

Finally, we validated the DSP profile using immunohistochemistry. The percentage of immunohistochemical staining in decidua and the DSP antigen counts in decidua were highly and significantly correlated for VISTA (r=0.95; p=8.1e-6; **Figure 5A**]. Cell staining for VISTA was widely expressed in chorionic macrophages in the saline control animals, which was verified by CD163 staining (**Figures 5B, C**). VISTA immunostaining was also significantly higher in the decidua in the GBSΔ*covR* group compared to other groups (**Figures 5D, E**).

### 3.2 Peak AF cytokine concentration correlated with immune checkpoint expression

To determine whether spatial profiles of expressed immunoproteins in the placental chorioamniotic membranes and maternal decidua are related with AF inflammatory proteins, we correlated AF cytokine concentrations with immunoprotein antigen counts in the amnion, chorion, and decidua. First, we evaluated the AF cytokine distribution in the samples for which we had digital spatial profiling data, which represented a subset of previously published data with an additional unpublished control (**Supplementary Figure 2**) (Boldenow et al., 2016). As expected, there was a significantly increased concentration of multiple inflammatory cytokines (IL-6, I-TAC, IL-8, IL-1β, MCP-1, TNF-α) in the GBSΔ*covR* group versus saline controls (all p<0.05).

**Figure 6:**
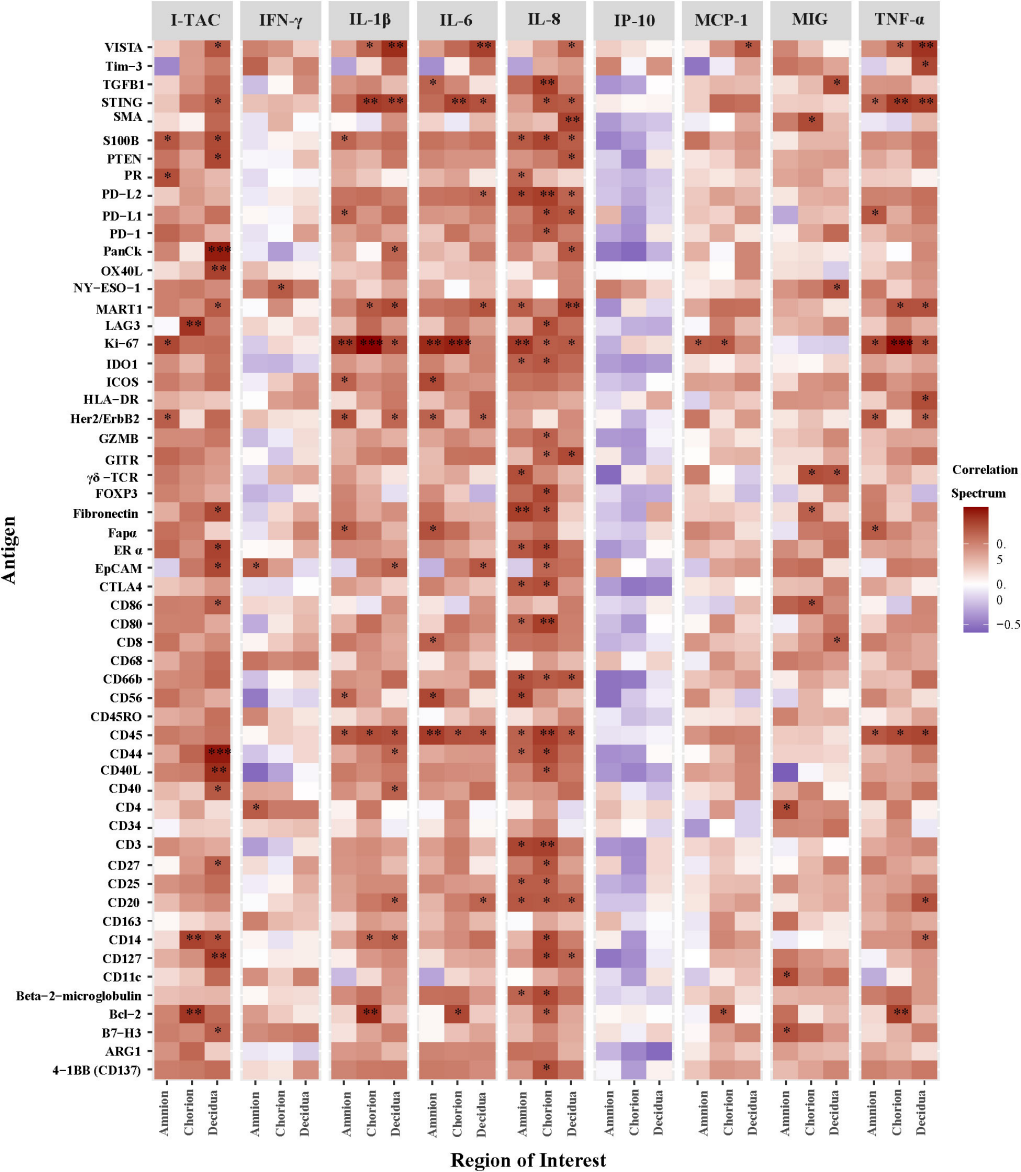
Heatmap of correlations between DSP antigen counts and amniotic fluid cytokine concentrations by Region of Interest. Log10 cytokine concentrations were correlated to log10 S6 normalized counts of immunoproteins (y-axis) for GBSΔ*cov*R, GBSΔ*covR*Δ*cylE* and controls. All 56 immunoproteins have been included, to evaluate the correlation in either the amnion, chorion or decidua using Spearman rank-order correlation (*, p <0.05; **, p<0.01; ***, p<0.001).

Next, we correlated AF cytokines with immune protein antigen counts expressed at the site of GBS inoculation within the amnion, chorion, and decidua (**Figure 6**). Among the ROI, chorion and decidua had the greatest number of correlations with peak AF cytokine concentrations. Of the immune checkpoint proteins in our DSP panel, VISTA had the greatest number of significant correlations with peak AF cytokine concentrations (I-TAC, MCP-1 IL-1β, IL-6, IL-8, and TNF-α) within either the chorion or decidua. Of the AF cytokines evaluated, IL-8 had the greatest number of significant correlations with overall DSP antigen counts and immune checkpoint proteins captured by the DSP (i.e., inhibitory: VISTA, PD-1/PDL-1/PDL-2, LAG3, CTLA4; stimulatory: GITR, CD27, CD40L CD137; p<0.05). There were strikingly different patterns of correlation between T cell chemokines and stimulatory/inhibitory immune checkpoint proteins. For example, peak AF concentration of I-TAC was highly and significantly correlated with multiple immune checkpoint antigens (inhibitory immune checkpoints: VISTA, B7-H3; stimulatory immune checkpoints: OX40L, LAG3, CD27, CD40/CD40L; all p<0.05). In contrast, there were few correlations between MIG and immune checkpoint antigens and none for IP-10. Overall, these findings reveal the selective and coordinated regulation of immune checkpoint proteins in placental chorioamniotic membranes with cytokine elevations in the amniotic fluid.

### 3.3 Uterine activity was moderately correlated with expression of few immunoproteins but not of immune checkpoints

**Figure 7:**
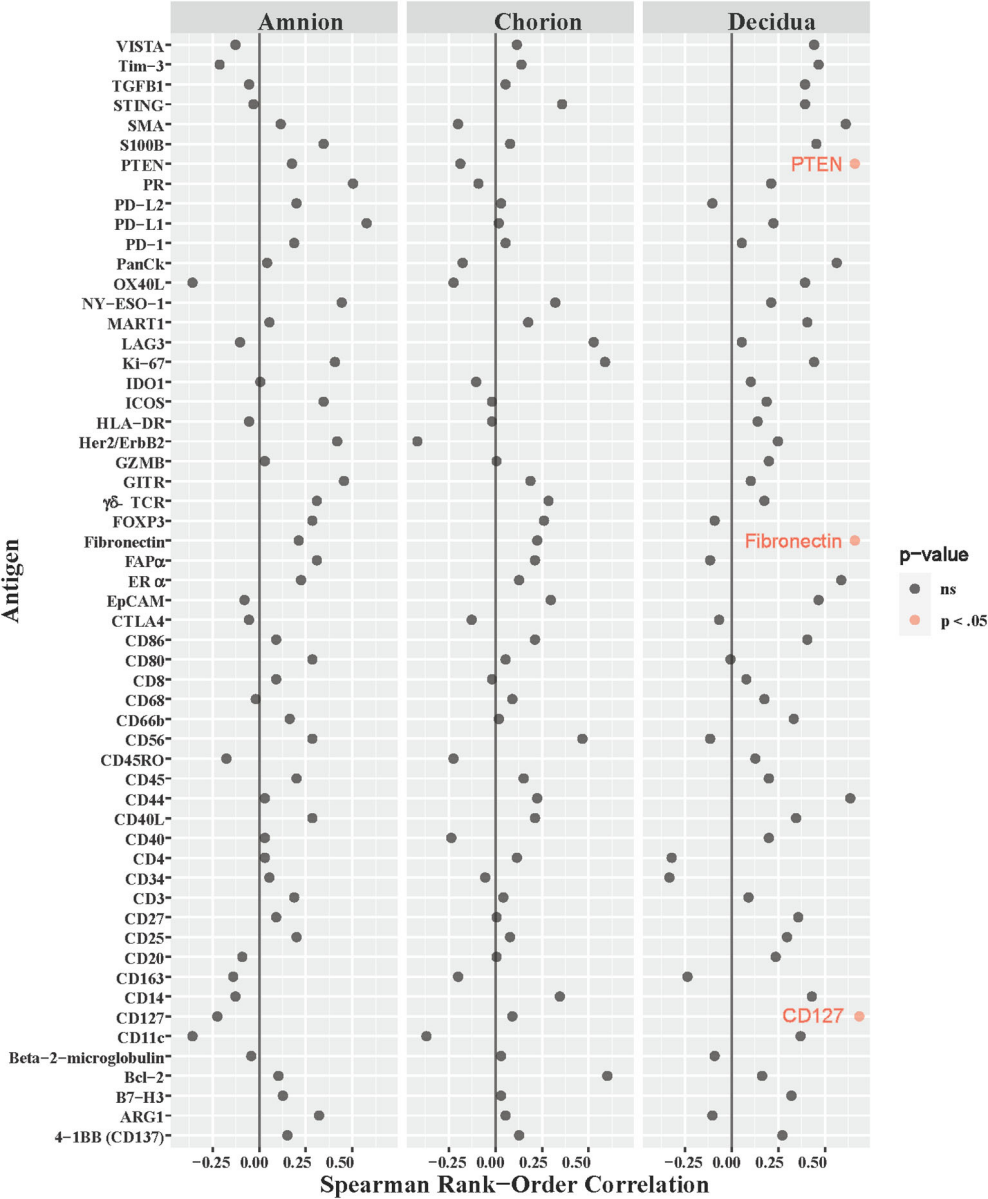
Correlations between DSP antigen counts and Peak 24-hour contraction area. The peak average hourly contraction area per day was correlated to log10 S6 normalized counts of immunoproteins (y-axis) for GBSΔ*cov*R, GBSΔ*covR*Δ*cylE* and controls. All 56 immunoproteins have been included, to evaluate the correlation in either the amnion, chorion or decidua using Spearman Rank order correlation coefficient. Black dots indicate non-significant (ns) antigens, while colored dots indicate antigens with a statistically significant p-value (light pink, p <0.05).

Our NHP model was designed to measure intraamniotic fluid pressure through implanted catheters to obtain quantitative and continuous measures of uterine contraction activity (Li et al., 1054; Adams Waldorf et al., 2011a; Adams Waldorf et al., 2011b; Boldenow et al., 2016). We asked whether uterine activity, measured by the peak hourly contraction area in 24 hours (**Supplementary Table 2**), correlated with immune checkpoint or other immunoprotein antigen counts in the amnion, chorion, and decidua. Of the 168 antigens analyzed across all three ROIs in the GBSΔ*covR* versus saline contrast, only the counts for three antigens had moderate correlations with peak hourly contraction area in the decidua: fibronectin (ρ=0.66, p=0.04), CD127 (ρ=0.68, p=0.035) and phosphatase tensin homolog (PTEN; ρ=0.66, p=0.04; **Figure 7**). There was no relationship between immune checkpoint protein expression and uterine activity.

### 3.4 Placental redline histopathologic scoring correlated with only inhibitory immune checkpoint expression

**Figure 8:**
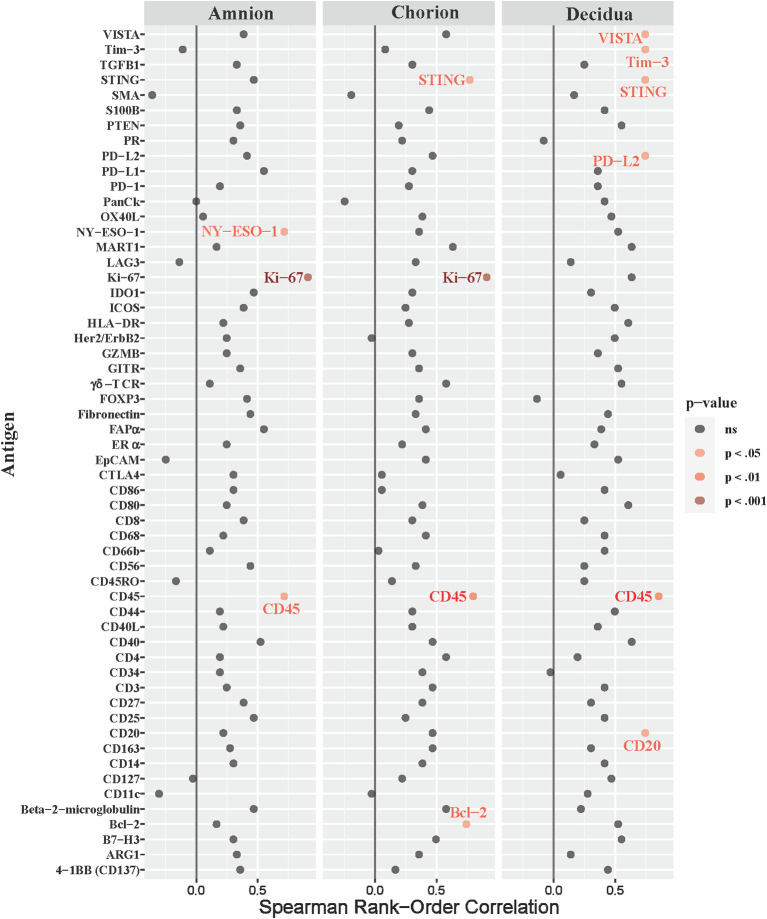
Correlations between DSP antigen counts and placental redline maternal stage score. Placental Redline Maternal Stage scores were correlated to log10 S6 normalized counts of immunoproteins (y-axis) for GBSΔ*cov*R, GBSΔ*covR*Δ*cylE* and controls. All 56 immunoproteins have been included, to evaluate the correlation in either the amnion, chorion or decidua using Spearman Rank order correlation coefficient. Black dots indicate non-significant (ns) antigens, while colored dots indicate antigens with a statistically significant p-value (light pink, p <0.05; medium pink, p<0.01; red, p<0.001).

Next, we asked whether immune checkpoint expression correlated with histopathological evidence of placental inflammation, as quantified by the Redline criteria (Redline et al., 2003). Redline scoring was performed by a pediatric and placental pathologist with focus on scores assigned to maternal stage and maternal grade (**Supplementary Table 2**). The maternal stage score reflects the duration of the inflammatory response based on the presence of neutrophils spread in segmented tissues, while the maternal grade score reflects the maternal and fetal inflammatory response based on the presence of abscesses in different tissue areas. When analyzing both maternal stage and grade, only inhibitory immune checkpoints (VISTA and Tim-3) were significantly correlated with either the stage or grade score in the chorion and decidua (maternal stage, **Figure 8**; **Table 3**; maternal grade, **Supplementary Figure 3**; all p<0.05). The maternal stage Redline score also correlated with PD-L2 expression in the decidua. No stimulatory immune checkpoints correlated with maternal stage or grade scores.

**Table 3:** Antigens significantly correlated with red maternal stage score.

Antigen	Amnion		Chorion		Decidua	
	ρ	p-value	ρ	p-value	ρ	p-value
Bcl-2	–	–	0.7	0.02	–	–
CD20	–	–	–	–	0.7	0.02
CD45	0.7	0.03	0.8	0.009	0.9	0.003
Ki-67	0.9	7 x 10^-4^	0.9	7 x 10^-4^	–	–
NY-ESO-1	0.7	0.03	–	–	–	–
PD-L2	–	–	–	–	0.7	0.02
STING	–	–	0.8	0.02	0.7	0.02
Tim-3	–	–	–	–	0.7	0.02
VISTA	–	–	–	–	0.7	0.02

The table shows the ρ (Spearman correlation coefficient) and p-value within each region of interest for each antigen that had a significant correlation.

## 4 Discussion

### 4.1 Summary of major findings

Immune checkpoints are critical for maintaining immunologic homeostasis and can be co-opted within tumors and during infection to facilitate immune evasion. In our nonhuman primate model, we could spatially profile the immune checkpoint expression at the maternal-fetal interface exposed to a GBS experimental infection. Our data indicates that an early and spatial immunologic response occurs in the placental chorioamniotic membranes and decidua, which upregulates expression of multiple inhibitory immune checkpoint proteins (VISTA, PD-L1, LAG3) and a few activating checkpoint proteins (OX40L, CD40/CD40L, GITR) during a GBSΔ*covR* inoculation. The lack of differentially expressed immune checkpoints in the placental membranes and decidua between saline and GBSΔ*covR*Δ*cylE* groups reflects the importance of β-hemolysin in driving immune checkpoint expression. The broad spectrum of stimulatory and inhibitory immune checkpoint proteins was highly, positively correlated with several AF inflammatory cytokines (e.g., IL-8) and the Redline maternal stage/grade scores indicating an association between these classes of immune proteins and the inflammatory response within the AF and at the maternal-fetal interface.

The inhibitory immune checkpoint VISTA had the highest differential expression in the decidua, which was due mainly to VISTA expression on infiltrating neutrophils. In contrast, there were relatively few significantly expressed immunoproteins in the amnion and chorion, underscoring the importance of decidual immune cells in the placental/maternal host response to infection. Overall, our data reveals the complexity of pro- and anti-inflammatory responses at the site of a GBS infection with an intriguing role for VISTA and other immune checkpoint proteins to modulate the maternal immune response during acute chorioamnionitis.

### 4.2 Role of immune checkpoints in normal and adverse pregnancy outcomes

The immunologic balance required to maintain tolerance to semi-allogeneic fetal cells is complex. Immune checkpoint proteins have previously been detected within gestational tissues and peripheral blood in normal and abnormal pregnancies. In the term placenta, immune checkpoints are expressed by a diverse repertoire of cells at the maternal-fetal interface including cytotrophoblast cells, stromal decidual cells, and decidual lymphocytes with a changing profile of expression across gestational age (Petroff and Perchellet, 2010). Investigation of immune checkpoints revealed elevated levels of soluble PD-L1 (sPD-L1) and Galectin-9 (Gal-9) in peripheral blood of pregnant women and placental chorionic villous tissues, suggesting that inhibitory immune checkpoint expression may be important for normal pregnancy (Enninga et al., 2018). Further studies suggested a low sPD-L1 may be a biomarker for spontaneous abortion (Li et al., 2023). Dysregulation of immune checkpoints have been associated with adverse pregnancy outcomes. Expression of immune checkpoints are also increased in the pro-inflammatory state of preeclampsia (PD-1/PD-L1 system, TIM-3, CD40/CD40L) (Matsubara et al., 2016; Herrock et al., 2023). During early stages of pregnancy, LAG-3 CTLA4 T+ regulatory cells have been found in the periphery and decidua and have been postulated to inhibit proliferation of tolerance against the fetus (Marozio et al., 2023). Dysregulation of immune checkpoints has also been associated with recurring pregnancy loss. Increased expression of immune checkpoints LAG-3 and CTLA4, and decreased expression of CD276 have been demonstrated to negatively affect implantation and contributes to overactivation of the immune system in the endometrium, preceding tissue of the decidua (Marozio et al., 2023). Data from this study indicate that immune checkpoint upregulation occurs during an early GBS infection within the placental chorioamniotic membranes and decidua, which may favor either the host or pathogen.

### 4.3 Role for inhibitory immune checkpoint proteins in GBS infection within chorioamniotic membranes

Multiple activating and inhibitory immune checkpoint proteins were upregulated in the placental chorioamniotic and decidua tissues after GBS inoculation (i.e., VISTA, PD-L1, LAG3, OX40L, GITR). Regulation of VISTA expression was a key finding in our study, as it demonstrated the highest difference between experimental groups and broadest correlation with AF cytokine concentration and maternal placental redline stage scoring of the immune proteins in the DSP panel. VISTA is an inhibitory immune checkpoint expressed primarily on myeloid derived immune cells, including macrophages, monocytes, neutrophils, and both lymphoid and myeloid subsets of dendritic cells and at lower levels on naïve CD4+ and CD8+ T cells (Lines et al., 2014). Studies have shown that deficiency of VISTA leads to dysregulation of macrophage chemotaxis due to downregulation of chemokine receptors (CCR2, CCR5, MCP-1 receptor) (Broughton et al., 2019). Chemotaxis of myeloid cells are crucial for host response during bacterial infection, which is partially regulated through expression of VISTA. After GBS infection with the hypervirulent strain (GBSΔ*covR*), VISTA immunostaining was significantly increased in the decidua with morphology suggestive of neutrophil and macrophage expression. The relationship between neutrophil influx and immune checkpoint expression in the decidua may be critical for defining the chemotactic response.

Immune checkpoint proteins control T cell activation and when expressed by neutrophils can impair the function of cytotoxic CD8+ T cells (van der Zwan et al., 2018). In humans, CD4+ and CD8+ T cells within the maternal decidua represent approximately 30-40% and 45-75% of leukocytes humans (Nancy and Erlebacher, 2014). Studies have demonstrated that both CD4+ and CD8+ T cells from human third trimester decidua have a highly differentiated effector memory phenotype and express higher levels of immune checkpoints (PD-1, TIM-3, CTLA-4 and LAG-3) in comparison to peripheral blood (Powell et al., 2017; van der Zwan et al., 2018). Our findings of the regulation of immune protein expression after GBS infection within the chorion and decidua may be important in understanding how decidual CD4+ and CD8+ T cells balance the role of preventing an allogeneic response against the fetus while protecting against infection (Taub et al., 1993; van der Zwan et al., 2018; Kohli et al., 2022).

The association of immune checkpoint expression with pro-inflammatory cytokine concentrations is comparable to literature in oncology describing a tumor microenvironment. A hallmark feature of our GBS NHP model is neutrophil influx into the decidua and chorioamniotic membranes with release of neutrophil extracellular traps after inoculation of GBSΔ*covR* (Boldenow et al., 2016). In tumor microenvironments, neutrophils infiltrate different cancers and express inhibitory immune checkpoints, including ARG1 and PD-1/PDL1, which restrict immune surveillance of malignant cells (Buddhisa et al., 2015; He et al., 2015; Moses and Brandau, 2016; Scapini et al., 2016). Chemotactic cytokines recruiting neutrophils (e.g., IL-8) into tumors have been investigated as potential prognostic markers for the effectiveness of cancer drugs targeting inhibitory immune checkpoints (de Andrea et al., 2021; Wang et al., 2021). In addition, neutrophil release of NETs within a tumor is also linked to secretion of immune checkpoint proteins, a finding associated with a poorer prognosis in several cancers including pancreatic ductal adenocarcinoma (Buddhisa et al., 2015; Boldenow et al., 2016; Demers et al., 2016; Moses and Brandau, 2016; de Andrea et al., 2021). Our data supports further studies to investigate the key role of immune checkpoint proteins in the immune response of an evolving infection in the chorioamniotic membranes and decidua. Whether immune checkpoints may promote pathogen survival or activate host responses is unknown.

### 4.4 Role for stimulatory immune checkpoint proteins in GBS infection within chorioamniotic membranes

Many stimulatory immune checkpoint proteins/ligands were significantly increased in the amnion, chorion and decidua, but primarily the decidua, in the GBSΔ*covR* versus saline group [GITR, OX40L, CD40, CD40L, tumor necrosis factor receptor superfamily (TNFRSF) receptors]. TNFRSF receptors are well characterized as proinflammatory markers that can bind TNFs, trigger cell death and are primarily expressed on myeloid and/or lymphocytes (Dostert et al., 2019; Liu et al., 2022). The corresponding ligands are found on a multitude of cells, including OX40L on antigen presenting cells and CD40L on activated T and B cells, platelets, monocytic cells, natural killer cells and basophils. Surprisingly, in our study TNFRSFR receptors and ligands did not correlate with AF TNF-α concentrations. In contrast to inhibitory immune checkpoints (e.g., VISTA), not one stimulatory immune checkpoint consistently correlated significantly in antigen counts with either AF cytokine concentrations, uterine activity or placental histopathology scores. This may suggest that the inhibitory immune checkpoint VISTA has a more consistent and greater change in expression, which could facilitate immune evasion by the bacteria.

### 4.5 Clinical and research implications

This study has provided insight into immune checkpoints in an acute bacterial infection at the maternal-fetal interface. The role of immune checkpoint proteins in determining activation of adaptive immune cells in normal pregnancy and chorioamnionitis is unknown. Late preterm birth is often characterized by infiltration of CD8+ T cells into the chorioamniotic membranes, a condition called chronic chorioamnionitis (Lee et al., 2013; Kim et al., 2015). Investigating the regional expression of immunoproteins is critical to understanding the innate and cellular response and developing therapeutics that can manipulate the natural immune response to prevent adverse pregnancy outcomes. It is possible that inhibitory immune checkpoints upregulated during bacterial-host pathogen interactions in the decidua may prevent T cell infiltration over a few days but could be overwhelmed if the bacteria persist. Further research is necessary to determine how stimulatory and inhibitory immune checkpoint proteins may exclude or allow maternal T cells to infiltrate the chorioamniotic membranes.

### 4.6 Strengths and limitations

Immune checkpoint proteins are potential therapeutic targets for improving pregnancy outcomes following an infectious encounter. These proteins have been demonstrated to be upregulated in other acute and chronic infections, such as malaria, listeria, HIV, and hepatitis B (Wykes and Lewin, 2018). However, there are no prior studies to our knowledge that reveal changes in immune checkpoint expression at the maternal-fetal interface during an infection. Therefore, our study is the first to correlate regional expression of immune checkpoint proteins after GBS infection within the chorion and decidua with inflammation, uterine activity and histopathologic changes in the placental chorioamniotic membranes. As best practices for data normalization are not yet established for this new technology, the extensive testing of different normalization methods and validation with data from other platforms is a study strength. Finally, interrogating correlations between the chorion, decidua, and AF cytokines revealed crosstalk between these compartments.

There are also several limitations to this study including the number of animals and immunoproteins analyzed. This panel focused mainly on adaptive immune checkpoints and lacked several immune checkpoint receptors or ligands that would allow us to evaluate an entire immune checkpoint axis (e.g., OX40-OX40L; GITR-GITRL). Other proteins of interest not in the panel include signal-regulatory protein-α (SIRPα), an inhibitory receptor expressed on myeloid phagocytes, and LILRB2, found on both innate (monocytes, macrophages, basophils, and DCs) and adaptive (CD4+ T cells) cells (Lentz et al., 2021). These limitations can be addressed using the latest version of Nanostring GeoMx® and targeting the whole transcriptome. Additionally, this data does not allow us to elicit the functions these immune proteins in the context of a GBSΔ*covR* infection, but instead highlights the differential regulation of multiple immune checkpoint proteins. Finally, this experiment has a modest sample size in each experimental group and should ideally be validated in a human cohort; given the small N and exploratory nature of the investigation, multiple hypothesis correction was not performed. However, a limited study of two immune checkpoints in the peripheral blood of pregnant women and chorionic villous tissues suggested higher expression of PDL-1 in syncytiotrophoblast cells that validates in part the dynamic nature of immune checkpoint expression in the placenta (Enninga et al., 2018). More studies on the role of immune checkpoints in the placenta following infection are warranted.

## 5 Conclusions

Pregnancy requires an intricate balance of tolerance to fetal allograft while being able to respond to infection. Our study findings reveal the complexity of the pro- and anti-inflammatory response at the site of a GBS infection in the placental chorioamniotic membranes and decidua, which underscores the importance of the decidua and chorion in the host response. Expression of VISTA, an inhibitory immune checkpoint, within the decidua may represent an important inhibitory immune checkpoint at the site of an infectious stimulus that alters pathogen response by other immune cells.

## Data availability statement

The raw data supporting the conclusions of this article will be made available by the authors, without undue reservation.

## Ethics statement

The animal study was approved by University of Washington Institutional Animal Care Use Committee. The study was conducted in accordance with the local legislation and institutional requirements.

## Author contributions

GM: Conceptualization, Investigation, Supervision, Writing – original draft, Writing – review & editing, Formal Analysis, Methodology, Validation. MC: Data curation, Investigation, Writing – original draft, Writing – review & editing. AO: Data curation, Investigation, Writing – original draft, Writing – review & editing. JM: Data curation, Formal Analysis, Investigation, Methodology, Software, Validation, Visualization, Writing – original draft, Writing – review & editing. AL: Investigation, Writing – review & editing. RK: Conceptualization, Investigation, Methodology, Validation, Writing – review & editing. ML: Investigation, Writing – review & editing. EL: Investigation, Writing – review & editing. BA: Formal Analysis, Investigation, Writing – review & editing. LR: Conceptualization, Formal Analysis, Funding acquisition, Investigation, Project administration, Supervision, Writing – original draft, Writing – review & editing. KA: Conceptualization, Formal Analysis, Funding acquisition, Investigation, Project administration, Resources, Supervision, Validation, Writing – original draft, Writing – review & editing.
