# Supplementary material for: Spatial profiling of the placental chorioamniotic membranes reveals upregulation of immune checkpoint proteins during Group B Streptococcus infection in a nonhuman primate model
Source: Front Cell Infect Microbiol. 2024 Jan 4;13:1299644. doi: 10.3389/fcimb.2023.1299644 (PMC10794649; doi:10.3389/fcimb.2023.1299644)
Supplement: Supplementary file 1 [file DataSheet_1.pdf]

Supplementary Material

Contents

1 Supplementary Methods 2

1.1 Data Normalization 2

2 Supplementary Tables 3

2.1 Table S1. Experimental Design for each Animal 3

2.2 Table S2. Uterine Activity, Clinical Outcomes, Placental Histopathology Score and Fetal Sex for Each Experiment 4

2.3 Table S3. Significantly Differentially Expressed Immunoprotein in the Decidua at the Site of a GBSΔ*covR*Δ*cylE* Inoculation 5

3 Supplementary Figures 6

3.1 Figure S1. Correlation of Tissue Area and GAPDH, Histone, and S6 Antigen Counts 6

3.2 Figure S2. Amniotic Fluid Cytokine Concentration in GBSΔ*covR* and Control Groups 7

3.3 Figure S3. Correlations Between DSP Antigen Counts and Placental Redline Maternal Grade Score 8

1 Supplementary Methods

1.1 Data Normalization

Several normalization strategies were modeled and validated against previously published immunohistochemical data. First, we determined the correlation between the provided aggregate normalization factor (including GAPDH, H3, S6) and tissue area normalization factor within the amnion, chorion, and decidua (Fig. S1). Normalization factors using an aggregate of the housekeeping proteins and tissue area were highly positively correlated (amnion, r=0.83; chorion, r=0.88; decidua, r=0.82). Unexpectedly, data normalization using the aggregate housekeeping factor yielded similar CD66b (neutrophil marker) expression between GBSΔ*covR* and saline controls; this finding conflicted with data from past studies revealing neutrophil infiltration in the GBS model consistent with acute chorioamnionitis. Therefore, we explored different methods to determine the most suitable normalization factor that could be validated using our existing data from diverse platforms in our model.

First, we determined the correlation between tissue area and antigen counts for each individual housekeeping protein (GAPDH, H3, S6) to understand if normalization to a single housekeeping protein might outperform normalization to other individual proteins or the aggregated data. The overall Pearson correlation coefficient values between tissue area and individual housekeeping proteins were strong (GAPDH, r=0.69; H3, r=0.7, S6, r=0.84; all p≤0.001; Fig. S1A). However, when normalization was performed within each ROI (amnion, chorion, decidua), correlation coefficients for GAPDH and H3 were weaker (r=0.23-0.49; Fig. S1B). In contrast, normalization to the ribosomal protein S6 within each ROI maintained a consistent strong correlation with tissue area (r=0.67-0.77; all p<0.02; Fig. S1B).

2 Supplementary Tables

2.1 Table S1. Experimental Design for each Animal

Group Name	Animal ID	Inoculation Amount (CFU)	Gestational Age at Necropsy (Days)	Interval Between Inoculation and Necropsy (Days)
Saline	A03140	N/A	139.4	7
Saline	L07201	N/A	139.4	7
Saline	M05062	N/A	155.4	91.5
Saline	M99183	N/A	143.4	7
GBSΔ*covR*	A10033	1.3 x 10^8	135.6	1
GBSΔ*covR*	A12263	5.6 x 10^8	130.6	0.25
GBSΔ*covR*	Z08140	4 x 10^8	146.4	1
GBSΔ*covR*	A10016	1.3 x 10^8	131.9	3
GBSΔ*covR*Δ*cylE*	A09114	5 X 10^8	132.4	3
GBSΔ*covR*Δ*cylE*	A10212	5 X 10^8	133.4	3
GBSΔ*covR*Δ*cylE*	A10219	5 X 10^8	138.5	3
GBSΔ*covR*Δ*cylE*	A10223	5 x 10^8	132.4	3

This table shows the experimental details for each individual animal which includes the inoculation amount, gestational age at necropsy, and interval between inoculation and necropsy.

2.2 Table S2. Uterine Activity, Clinical Outcomes, Placental Histopathology Score and Fetal Sex for Each Experiment

Group Name	Animal ID	Peak Average Hourly Contraction [(mmHg·sec)/ hr]	Maternal Stage	Maternal Grade	Microbial Invasion of the Amniotic Cavity	Preterm Labor	Fetus Sex
Control	L07201	242.17	N/A	N/A	No	No	Male
Control	M99183	325.63	0	0	No	No	Male
Control	A03140	2747.12	N/A	N/A	No	No	Female
Control	M05062	N/A	0	0	No	No	Female
GBSΔ*covR*	A12263	7298.72	1	2	Yes	Yes	Male
GBSΔ*covR*	A10016	3522.29	3	2	Yes	Yes	Female
GBSΔ*covR*	Z08140	1417.12	N/A	N/A	Yes	Yes	Male
GBSΔ*covR*	A10033	6586.53	2	2	Yes	Yes	Female
GBSΔ*covR*Δ*cylE*	A10212	2372.80	0	0	No	No	Male
GBSΔ*covR*Δ*cylE*	A10223	2818.25	0	0	No	Yes	Female
GBSΔ*covR*Δ*cylE*	A10219	2254.38	0	0	No	No	Male
GBSΔ*covR*Δ*cylE*	A09114	N/A	2	1	No	No	Male

This table shows uterine activity peak hourly contraction area (mean 24-hour), maternal stage and grade, and clinical outcomes including presence of microbial invasion for the amniotic cavity, sex of the fetus, and preterm labor for each animal. Uterine activity was not obtained in two animals due to instrument malfunction after catheterization. Placental Redline score was not obtained in three cases.

2.3 Table S3. Significantly Differentially Expressed Immunoproteins in the Decidua at the Site of a GBSΔ*covR*Δ*cylE* Inoculation

Antigen	Mean Expression for Saline Controls	Mean Expression for GBSΔ*covR*Δ*cylE*	Pooled Standard Deviation	Standard Differences between GBSΔ*covR*Δ*cylE* and Saline Controls	Kruskal-Wallis Statistic	Kruskal-Wallis p-value
Decidua						
$\gamma\delta$-TCR	1.19	1.56	0.17	2.24	5.33	0.020

This table lists the significantly differentially expressed immunoprotein within the decidua for the GBSΔ*covR*Δ*cylE* versus saline group.

3 Supplementary Figures

3.1 Figure S1. Correlation of Tissue Area and GAPDH, Histone, and S6 Antigen Counts

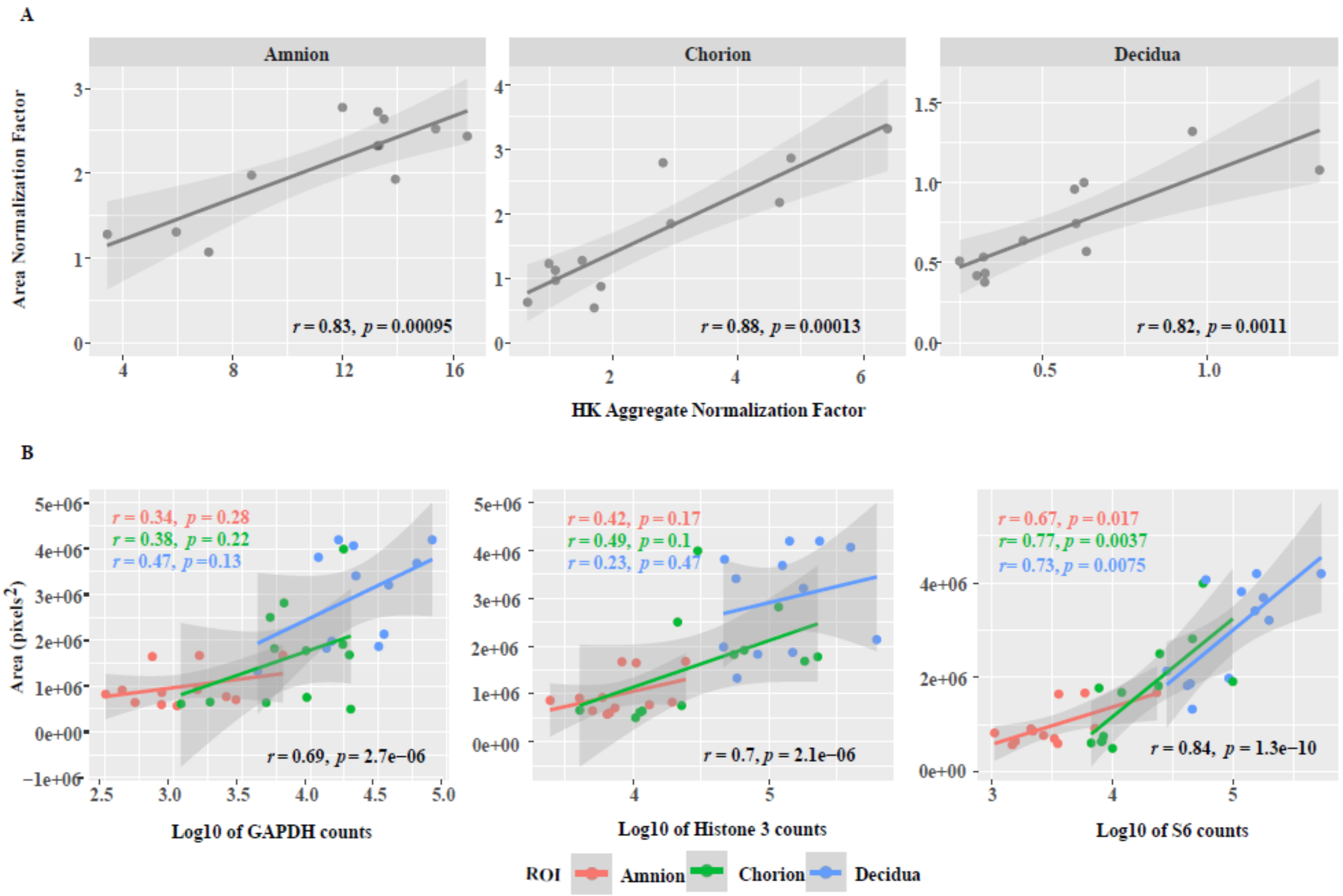

(A) The graph shows the correlation between tissue area and the housekeeping normalization factors provided by Nanostring GeoMx® in the amnion, chorion, and decidua. **(B)** The graph shows how the counts of individual housekeeping antigens (GAPDH, Histone, S6) correlate with the tissue area in the amnion (red), chorion (green), and decidua (blue). The overall line of best fit was labelled in black. The Pearson correlation test was used to calculate the correlation coefficient ρ (or R) value for different normalization strategies using individual housekeeping genes. P-values are located on the graphs.

3.2 Figure S2. Amniotic Fluid Cytokine Concentration in GBSΔ*covR* and Control Groups

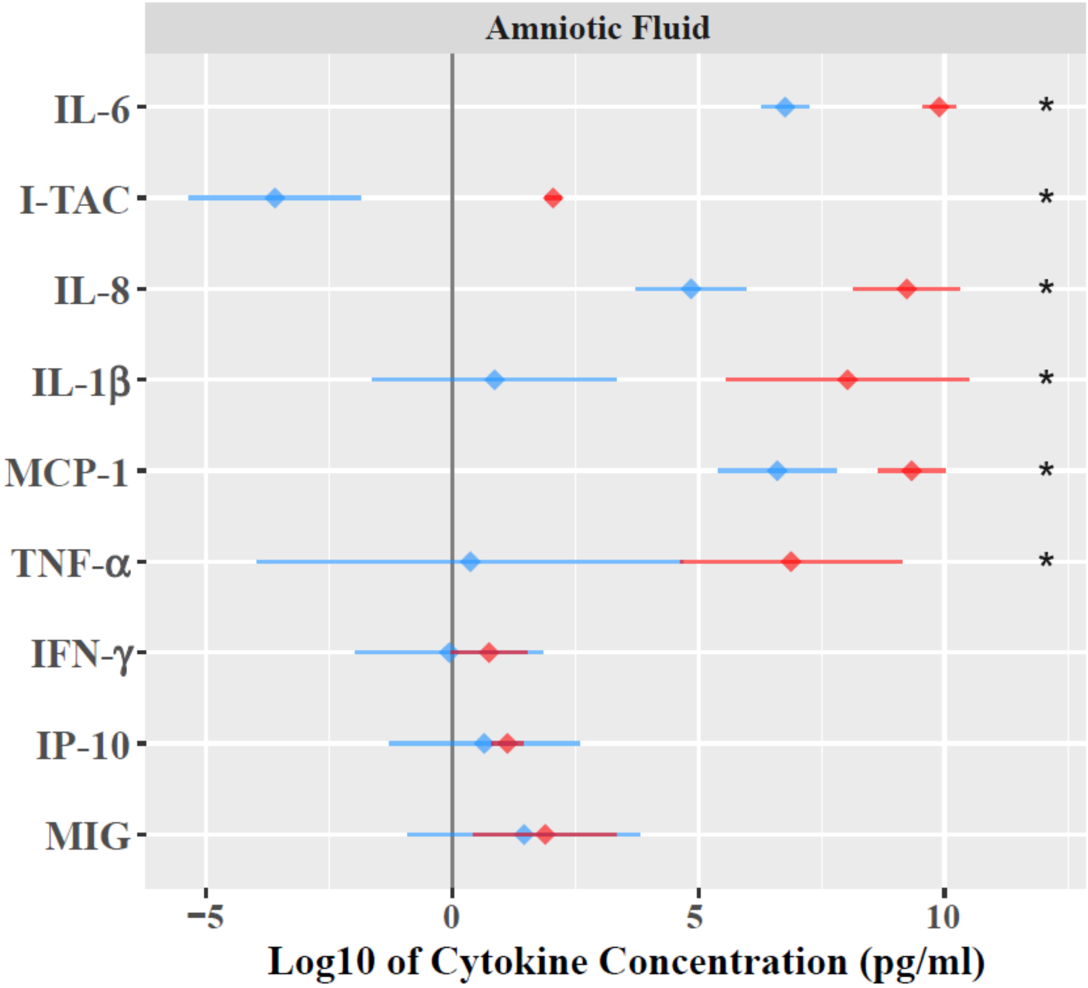

The log10 cytokine/ chemokine concentration (pg/mL) in amniotic fluid is shown on the x- axis for GBSΔ*covR* (red) and saline controls (blue). (*, $p < .05$)

3.3 Figure S3. Correlations Between DSP Antigen Counts and Placental Redline Maternal Grade Score

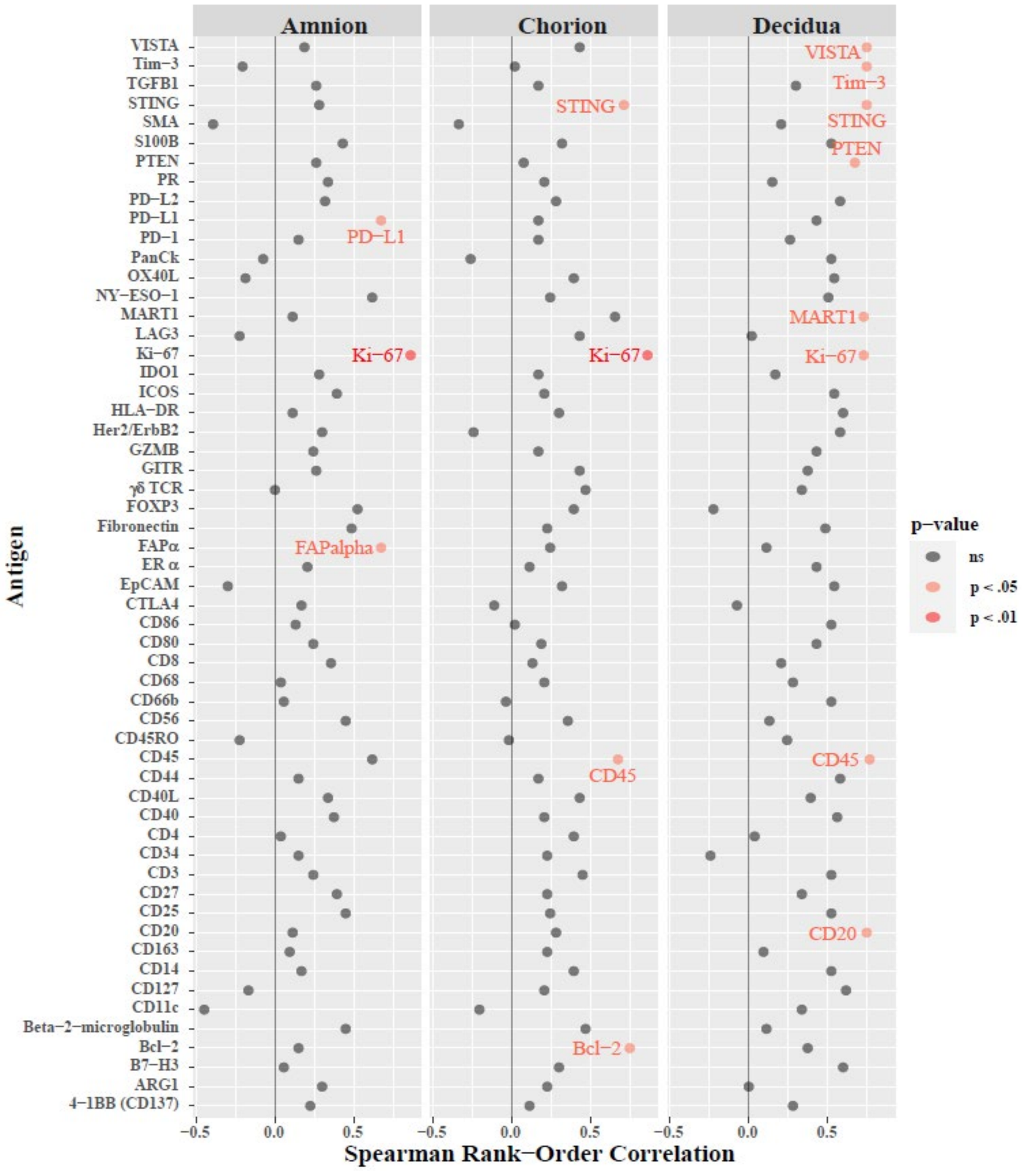

In this plot, the placental Redline maternal stage scores were correlated to the log10 S6 normalized antigen counts for each immunoprotein (y-axis) for GBSΔcovR, GBSΔcovRΔcylE and controls. All 56 immunoproteins have been included to show the presence or absence of a significant correlation in either the amnion, chorion or decidua using Sperman Rank order correlation coefficient. Black dots indicate non-significant (ns) antigens, while colored dots indicate antigens with a statistically significant p-value (light pink, $p < 0.05$; medium pink, $p < 0.01$; red, $p < 0.001$).
